# Effect of repetitive peripheral magnetic stimulation for patients with chronic stroke: a case report using an AB design

**DOI:** 10.3389/fresc.2025.1617492

**Published:** 2025-08-01

**Authors:** Shota Itoh, Kenta Fujimura, Shogo Imamura, Ryoka Itoh, Yuma Misawa, Mii Matsuda, Chisato Chikamori, Hiroki Tanikawa, Hirofumi Maeda, Hitoshi Kagaya

**Affiliations:** ^1^ Department of Rehabilitation, Fujita Health University Hospital, Toyoake, Aichi, Japan; ^2^ Faculty of Rehabilitation, School of Health Sciences, Fujita Health University, Toyoake, Aichi, Japan; ^3^ Department of Rehabilitation Medicine, School of Medicine, Fujita Health University, Toyoake, Aichi, Japan; ^4^ Department of Rehabilitation, National Center for Geriatrics and Gerontology, Obu, Aichi, Japan

**Keywords:** magnetic stimulation therapy, muscle spasticity, stroke, upper limb, neuromuscular disorders

## Abstract

**Objective:**

To determine the effects of repetitive peripheral magnetic stimulation in a patient with chronic stroke.

**Design:**

Case report.

**Patients:**

A man in his 70s presented with left hemiplegia secondary to cerebral hemorrhage.

**Methods:**

An AB design was used: phase A (sham stimulation) and phase B (active stimulation). Magnetic stimulation was applied using a peripheral magnetic stimulator (Pathleader™, IFG, Sendai, Japan). Outcomes were assessed at four points: before the intervention, after phase A, after phase B, and at follow-up (3 weeks after phase B) using the Modified Ashworth Scale, range of motion, Fugl-Meyer Assessment, Simple Test for Evaluating Hand Function, and Canadian Occupational Performance Measures.

**Results:**

The Modified Ashworth Scale score for the wrist extensor remained unchanged in phase A but improved after phase B and was sustained at follow-up. The range of motion showed no change. The Fugl-Meyer Assessment scores were 40, 41, 44, and 45, respectively, and the Simple Test for Evaluating Hand Function scores were 1, 4, 3, and 5, respectively, at the four time points. One Canadian Occupational Performance Measure item improved after phase B and remained stable.

**Conclusion:**

In patients with chronic stroke and severe hemiplegia, repetitive peripheral magnetic stimulation may be effective in reducing spasticity and improving motor function.

## 1 Introduction

Upper limb dysfunction following stroke significantly affects activities of daily living (ADL) and quality of life, presenting a major challenge to rehabilitation (1). Neuromuscular electrical stimulation (NMES) was developed to improve motor function in the paretic upper limbs of patients with stroke, and studies have demonstrated its efficacy in enhancing both upper limb motor ability and ADL performance (2, 3). However, NMES has several limitations, including stimulation-induced pain and time-consuming electrode placement procedures, as it requires surface electrodes for stimulus delivery.

Recent research has documented the therapeutic application of repetitive peripheral magnetic stimulation (rPMS), which repeatedly stimulates peripheral nerves and muscles to improve motor function and spasticity. Peripheral magnetic stimulation allows the preferential activation of deep motor and proprioceptive neural structures while circumventing cutaneous nociceptive afferents (4). It has been reported that when the same joint movement is produced by NMES and peripheral magnetic stimulation, the latter causes less pain than the former (5).

Several reports have demonstrated the therapeutic efficacy of rPMS in improving upper extremity motor function as measured using the Fugl-Meyer Assessment (FMA) (6) in patients with acute and subacute post-stroke hemiparesis (7, 8). In contrast, a meta-analysis of randomized controlled trials investigating the efficacy of rPMS for chronic post-stroke hemiparesis revealed that the time of post-stroke onset significantly affects therapeutic outcomes. The analysis demonstrated no significant improvements in motor function among patients in the chronic phase, suggesting a temporal dependency on therapeutic effectiveness (9).

Furthermore, although rPMS has a certain effect on improving proximal muscle function, there is insufficient evidence of its efficacy in improving distal muscle function. No unified conclusion has been reached regarding its effectiveness in terms of ADL (10). Therefore, the effects of rPMS applied to the distal muscles on upper extremity motor function and activities of daily living in patients with chronic stroke have not been sufficiently reported.

This study aimed to expand the evidence on the therapeutic effects of rPMS on the distal upper muscles of a patient with chronic stroke and hemiplegia. Using an AB design, we investigated the effects of rPMS applied to the distal upper muscles on motor function and activities of daily living in a single patient with chronic stroke.

## 2 Case description

The participant was a male patient in his 70s. The time since stroke onset following intracerebral hemorrhage was 4,613 days (approximately 12 years and 7 months). Because he could raise his paralyzed arm only to his nipple level and the finger separation movement was poor, his upper arm paralysis was assessed as 2 points on the Knee-Mouth test and 1C on the Finger test of the Stroke Impairment Assessment Set (SIAS) (11). He had been receiving botulinum toxin injections for upper limb spasticity every year. In this report, rPMS was introduced 6 months after botulinum toxin treatment to improve upper limb motor function.

## 3 Methods

### 3.1 Procedures

This study was approved as specified clinical research and registered with the Japan Registry of Clinical Trials (registration number: jRCTs042180062). Written informed consent was obtained from the participant. This study employed an AB design consisting of three phases: phase A (sham stimulation), phase B (active stimulation), and a follow-up phase, each lasting 3 weeks. During phases A and B, stimulation was administered three times per week for a total of nine sessions. Throughout both phases, the patient received 40 min sessions of both physical and occupational therapy during outpatient rehabilitation. Physical therapy consisted of stretching exercises and gait training for the affected lower extremities, whereas occupational therapy consisted of stretching exercises and object manipulation training for the affected upper extremities.

### 3.2 Outcome measures

**Figure 1:**
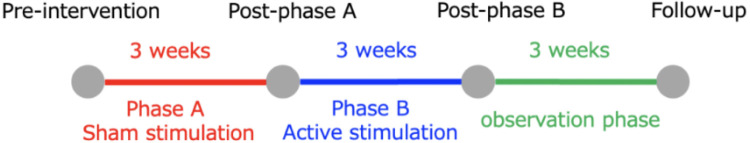
Research protocol. Assessments were conducted before intervention, after phase A, after phase B, and after the observation phase. Phase A (sham stimulation) and phase B (active stimulation), followed by the observation phase, were set at 3 weeks each. The intervention was performed thrice a week in phases A and B.

All assessments were conducted by an occupational therapist using a single-blind approach to accurately evaluate the effects of the intervention. Outcomes were measured at four time points: before the intervention, after phase A, after phase B, and at follow-up (3 weeks after phase B). The timing of each assessment was standardized to enable a detailed analysis of the effects of the intervention (Figure 1). Outcomes were assessed using the Modified Ashworth Scale (MAS) (12), range of motion (ROM), FMA, Simple Test for Evaluating Hand Function (STEF) (13), and Canadian Occupational Performance Measure (COPM) (14). The STEF was originally developed in Japan and is commonly used to assess hand function in stroke patients (13). In this test, patients are required to grip or pick up 10 objects of various shapes and sizes and transport them to a designated target. The 10 subtests consist of three spheres (large, medium, and small), rectangles, two cubes (medium and small), two small disks (wooden and metal), thin pieces of cloth, and pins. The transportation of each item is scored on a 10-point scale based on the time required, with a score of 0 assigned if the time limit is exceeded.

### 3.3 rPMS therapy

**Figure 2:**
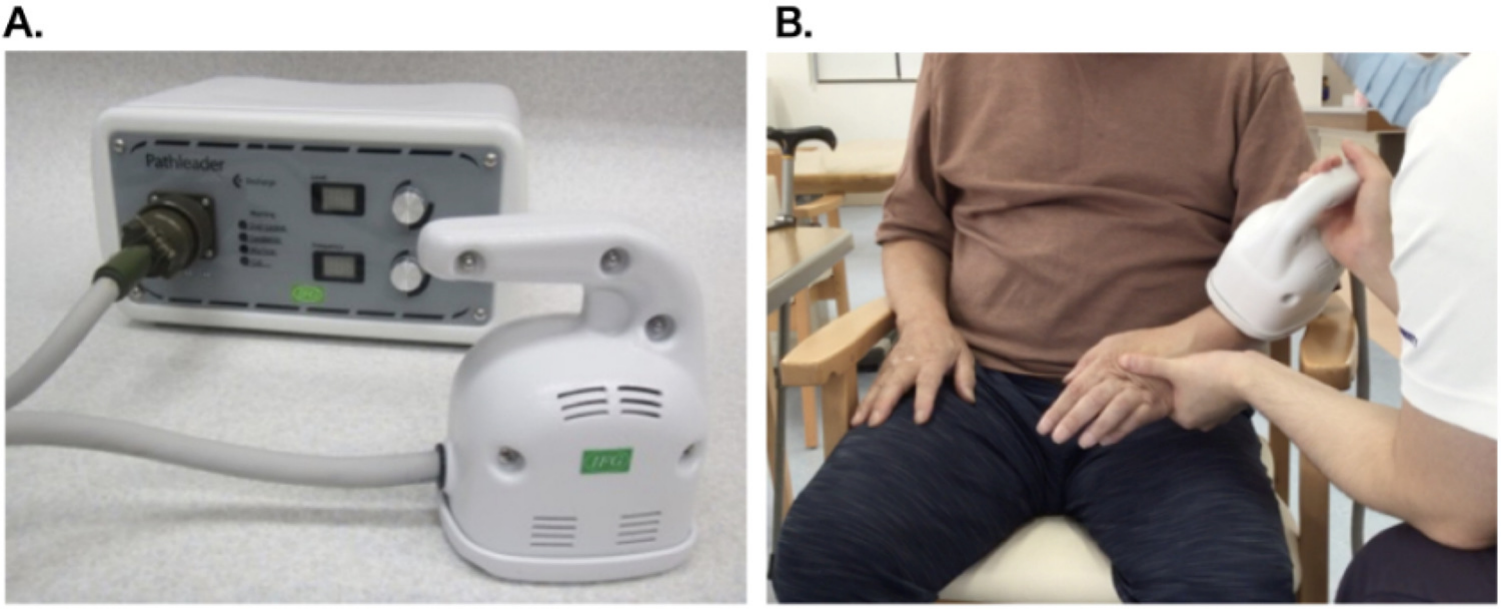
Magnetic stimulation device and intervention scene. **(A)** Magnetic stimulator: Pathleader™, **(B)** Intervention scene.

We used a commercially available peripheral magnetic stimulator (Pathleader^TM^, IFG, Sendai, Japan) for the rPMS treatment. The stimulation parameters were set to a frequency of 30 Hz, an intensity level of 80 (approximately double the patient's motor threshold), and an on/off ratio of 2 s/3 s. These parameters were determined based on previous clinical studies that demonstrated both efficacy and tolerability in the treatment of upper limb spasticity (7, 8), as well as in accordance with the manufacturer's guidelines. Each rPMS session lasted approximately 20 min, delivering a total of 6,000 pulses (100 trains of 60 pulses each). During both the sham and active sessions, the participant was instructed to actively perform voluntary wrist dorsiflexion and finger extension movements in synchrony with the auditory cues of the device. Stimulation was applied to the extensor digitorum communis and extensor carpi muscles on the dorsal aspect of the forearm. The intensity was adjusted to elicit wrist and finger extensions, and the therapist stimulated the patient's wrist in a neutral position to make it easier to extend the fingers (Figure 2). Sham stimulation was performed using the same coil placement and identical nominal settings (frequency and intensity) as in the active condition. However, the device was programmed to emit only the clicking sound without delivering an actual magnetic pulse. This approach ensured similar auditory and procedural experiences while eliminating physiological stimulation.

## 4 Results

The detailed results are shown in Table 1.

**Table 1:** Changes in outcome measures in each phase.

Outcome measures		Before intervention	After phase A	After phase B	Follow-up
MAS (points)	Wrist extensor muscles	1	1	0	0
	Wrist flexor muscles	0	0	0	0
ROM (degrees)	Wrist dorsiflexion	80	80	80	80
	Wrist flexion	30	30	30	30
FMA upper limb (points)	Total	40	41	44	45
	A	24	24	26	27
	B	6	6	6	6
	C	10	11	12	12
	D	0	0	0	0
STEF	Total score	1	4	3	5
	Number of items available for implementation	1	1	3	4
COPM (satisfaction/performance)	Total	15/14	16/15	19/18	19/20
	Opening and closing a bag	6/6	7/7	8/7	8/8
	Putting papers in and out	3/5	3/5	5/6	5/7
	Fasten a button on a shirt	6/3	6/3	6/5	6/5

MAS, modified Ashworth scale; ROM, range of motion; FMA, Fugl-Meyer assessment; STEF, simple test for evaluating hand function; COPM, Canadian occupational performance measure.

*MAS*: The wrist extension muscle score was 1 before the intervention and decreased to 0 after phase B; the effect was sustained until follow-up.

*ROM*: No changes were observed in any of the phases, including phases A and B, or at the follow-up.

*FMA*: The scores were 40, 41, 44, and 45 points before the intervention, after phase A, after phase B, and at follow-up, respectively. Improvements were observed after phase B and were maintained throughout the follow-up phase.

*STEF*: The number of items transported within the time limit increased to 1, 1, 3, and 4, whereas the total score changed to 1, 4, 3, and 5.

*COPM*: The three items listed were “opening and closing a bag”, “putting paper in and out”, and “fastening a button on a shirt”. For “opening and closing a bag”, satisfaction scores changed from 6 to 7, 8, and 8, while performance scores changed from 6 to 7, 7, and 8. For “putting papers in and out”, satisfaction scores changed from 3 to 3, 5, and 5, while performance scores changed from 5 to 5, 6, and 7. For “fasten a button on a shirt”, satisfaction scores remained at 6 across all assessments, while performance scores changed from 3 to 3, 5, and 5. The total satisfaction score changed from 15 to 16, 19, and 19, whereas the total performance score changed from 14 to 15, 18, and 20. The participant reported increased confidence and ease when performing daily activities that previously required assistance.

## 5 Discussion

In the present study, we used an AB design to examine the effects of rPMS in a patient with chronic stroke hemiplegia. Although no changes were observed during the sham stimulation phase, improvements were noted in several parameters following active stimulation. Since the participant was aware that he was receiving an intervention, the placebo effect cannot be completely ruled out. However, the observation that improvement occurred only after active stimulation suggests a specific effect of rPMS beyond placebo-related responses. Although the patient continued to receive routine outpatient physiotherapy and occupational therapy throughout all phases, no functional gains were documented prior to the intervention, indicating a plateau. Notably, improvements were observed exclusively during and after active rPMS, implying that these enhancements were primarily attributable to rPMS rather than to conventional therapy alone. Furthermore, although the patient had received botulinum toxin injections 6 months prior to the study, the clinical effects of such treatments typically persist for only 3–4 months. Therefore, we considered any residual impact on the functional outcomes to be minimal.

rPMS has been reported to improve the degree of spasticity, as evaluated using the Ashworth scale and MAS (15). Generally, NMES aims to reduce spasticity by applying electrical stimulation to the antagonist muscles of the spastic muscles, inducing muscle contractions based on the principle of reciprocal inhibition (16). In contrast, Transcutaneous Electrical Nerve Stimulation (TENS) has been reported to reduce spasticity by applying stimuli at intensities above the sensory threshold but below the motor threshold for the spastic muscles (17, 18). Agonist muscle stimulation can be used to enhance recurrent inhibition as an inhibitory pathway for agonist muscles. This is thought to be mediated by Renshaw cells, which provide negative feedback to *α*-motoneurons (19, 20). Based on these findings, in this study, we applied stimulation directly to the spastic muscles, consistent with previous studies that have used this approach. Although sham stimulation did not change the MAS score, it decreased after active stimulation, suggesting that rPMS may contribute to reduced spasticity in the stimulated muscle. The results of this study support previous reports that rPMS applied to the agonist muscle reduces the H/M ratio in healthy individuals (20) and spasticity in the wrist and finger flexors in patients with chronic stroke and CNS lesions (21). This is thought to support the mechanism by which rPMS acts directly on spastic muscles. However, we acknowledge that no neurophysiological assessments [e.g., H/M ratio and electromyography (EMG)] were performed in this study. Incorporating such measures into future research would be valuable for further elucidating the underlying mechanisms of rPMS, particularly its role in modulating recurrent inhibition and spinal excitability.

Before the intervention, the FMA score was 40/66, and the severity of upper limb function was classified as moderate paralysis (22). The minimal clinically important difference (MCID) for the FMA is 4 points (23), and the minimal detectable change is 3.2 points (24). Improvements were observed between the post-phase A period and the follow-up phase. This suggests that rPMS may contribute to the improvement of upper limb motor function in patients with stroke and moderate motor paralysis. These results suggest that it is possible to improve motor function, even in the chronic phase, with appropriate stimulation conditions and intervention frequencies. This addresses a notable gap in the literature. We interpret this as a possible cumulative or delayed effect, suggesting that neuromuscular adaptations may have continued beyond the intervention period under ongoing rehabilitation conditions.

The STEF score was low prior to the intervention, with the patient being able to perform only a few simple actions. Following active stimulation, the number of successfully completed items increased, accompanied by improvements in FMA scores. To our knowledge, no study has formally established minimal detectable change (MDC) or MCID values for the STEF. Therefore, it is impossible to conclude that the observed improvement represents a clinically meaningful change. However, the increase in STEF scores, when considered alongside improvements in other objective (e.g., FMA) and subjective (e.g., COPM) outcomes, suggests a broader trend toward enhanced functional use of the upper limb.

In this study, the MCID for the COPM was reported to be 2 points (14), and changes in the MCID were confirmed after active stimulation. The patient reported increased confidence and ease in daily activities, such as opening bags and managing paperwork, which previously required assistance. These improvements contributed to greater independence and enhanced participation in daily life, reflecting meaningful functional gains. Previous studies have suggested that changes in COPM satisfaction are more closely associated with improvements in fine hand function than with gross motor changes (25), which supports our observations.

In a previous report analyzing the improvement in upper limb function in patients with chronic stroke, the greatest improvement in upper limb function in patients with stroke occurred within 9 months of stroke onset (26), and the peak of improvement occurred within 14 weeks (27). Additionally, a 3-year follow-up study reported that no functional improvement was achieved 3 months after onset (28). Therefore, it is generally thought that it is difficult to achieve functional improvement in the upper limbs of patients with chronic stroke after 3 months. In contrast, in this case, more than 12 years had passed since the disease onset. Nevertheless, the introduction of rPMS led to improvements in the patient's upper limb motor function and ADL. This suggests that even in the chronic phase, frequent interventions three times a week can lead to improvements in upper limb function. We believe that these findings complement those of previous studies (29, 30).

## 6 Conclusions

rPMS can improve motor function, spasticity, and ADL in patients with chronic stroke hemiplegia.

## Data Availability

The original contributions presented in the study are included in the article/Supplementary Material, further inquiries can be directed to the corresponding author.
